# The *in vitro* Effect of Irrigants with Low Surface Tension on *Enterococcus faecalis*

**DOI:** 10.7508/iej.2015.03.006

**Published:** 2015-07-01

**Authors:** Luciano Giardino, Carlos Estrela, Luigi Generali, Zahed Mohammadi, Saeed Asgary

**Affiliations:** a *Researcher, Crotone, Italy; *; b * Department of Stomatological Sciences, Dental School, UFG-Federal University of Goiás, Goiânia, GO, Brazil; *; c * Dipartimento Chirurgico, Medico, Odontoiatrico e di Scienze Morfologiche con Interesse Trapiantologico, Oncologico e di Medicina Rigenerativa, Università di Modena e Reggio Emilia, Modena, Italy;*; d * Iranian Center for Endodontic Research (ICER), Research Institute of Dental Sciences, Dental School, Shahid Beheshti University of Medical Sciences, Tehran, Iran*

**Keywords:** *Enterococcus faecalis*, Hypoclean, Sodium Hypochlorite, Tetraclean

## Abstract

**Introduction:**

Due to the complex anatomy of the root canal system and high surface tension of common root canal irrigants (RCI), conducting an investigation on RCIs containing surfactants is a priority. The aim of this *in vitro* study was to verify the antibacterial potential of RCI with low surface tension in root canals infected with *Enterococcus faecalis* (*E. faecalis*).

**Methods and Materials:**

Thirty-five extracted human maxillary anterior teeth were prepared and inoculated with *E. faecalis* for 60 days. After root canal preparation, the teeth were randomly divided to one positive and one negative control groups and 5 experimental groups: Hypoclean/Tetraclean NA, Hypoclean, Tetraclean, NaOCl/Tetraclean and NaOCl. Bacterial growth was observed by turbidity of culture medium and then measured using a UV spectrophotometer. Data were analyzed in three time intervals (pre-instrumentation and, 20 min and 72 h after canal preparation) using the ANOVA and post hoc Tukey’s tests. The level of significance was set at 0.05.

**Results:**

The results indicated the presence of *E. faecalis* in all post-irrigation samples irrespective of the RCI. However, the optical densities in both post-irrigation periods showed bacterial reduction and significant differences between groups.

**Conclusion:**

RCI with low surface tension showed antibacterial potential in *E. faecalis *infected roots.

## Introduction

The causative role of microorganisms in the initiation and progression of pulp and periapical diseases is the proved fact in Endodontics [1-3]. Elimination of microorganisms from the infected root canal system is a complicated task involving the use of various instrumentation techniques, irrigation regimens and sometimes intracanal medicaments. Mechanical instrumentation alone does not guarantee a bacteria-free root canal system especially considering its complexity [4]. On the other hand, *in vitro* and clinical evidences have shown that mechanical instrumentation leaves significant portions of the root canal walls untouched [5] and complete elimination of the bacteria only through mechanical instrumentation is unlikely [6]. Any pulp tissue left in the root canals can potentially serve as a nutrient source for the remaining microorganisms. Furthermore, tissue remnants also impede the antimicrobial effects of root canal irrigants and medicaments [7]. Therefore, specific root canal irrigants (RCI) with tissue-dissolving and antibacterial abilities are necessary. 

Sodium hypochlorite (NaOCl) is the most common RCI with excellent antibacterial and tissue dissolving abilities. One of the major drawbacks of NaOCl is the high surface tension, that limits its penetration into irregularities of the root canal system such as fins, isthmi and dentinal tubules [8]. Adding a surfactant to NaOCl can resolve this problem. 

**Figure 1:**
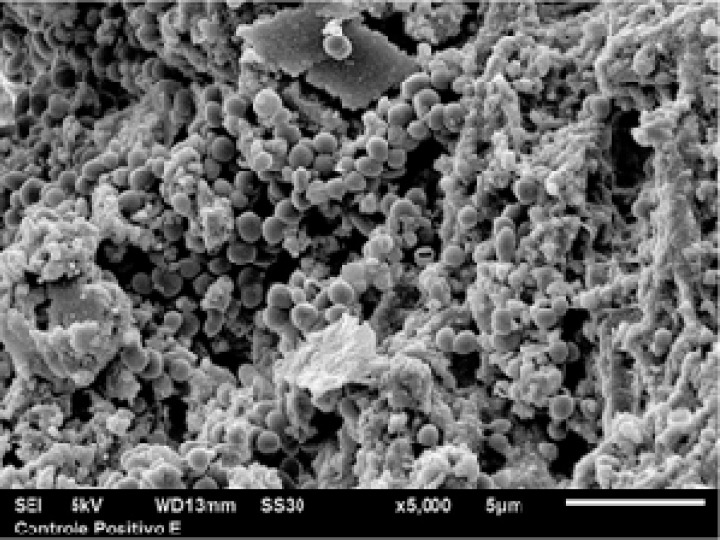
A scanning electron microscopy image of 60-day positive control samples with *Enterococcus Faecalis* (5000× magnification

Hypoclean (Ogna Laboratori Farmaceutici, Milano, Italy) is a detergent-based endodontic irrigant composed of 5.25% NaOCl and two detergents (cetrimide and polypropylene glycol) [9]. There are very few studies on the antibacterial activity of Hypoclean [9-12]. Hypoclean keeps its substantivity for up to 4 weeks [11]. Recently, it has been revealed that Hypoclean was the most effective agent against *Candida. albicans*, *Pseudomonas aeruginosa* and *Lactobacillus casei, in vitro *[12]. Tetraclean (Ogna Laboratori, Farmaceutici, Milano, Italy) is a new combination that contains doxycycline and detergents (citric acid and polypropylene glycol) [13-15]. Like Biopure MTAD (Dentsply, Tulsa Dental, Tulsa, OK, USA) which contains tetracycline isomer, citric acid and a detergent, Tetraclean is designed primarily for smear-layer removal with added antimicrobial activity. Both MTAD and Tetraclean contain citric acid, an antibiotic from tetracycline line and detergents [16]. They differ from each other in the concentration of citric acid and type of the detergents included [17]. They do not dissolve the organic tissue and are intended for use at the end of chemomechanical canal preparation subsequent to using NaOCl. Although earlier studies showed promising antibacterial effects by MTAD [18, 19], recent studies have indicated that the subsequent use of NaOCl and 17% ethylenediaminetetraacetic acid (EDTA) is equally or more effective than NaOCl/MTAD [20, 21]. Comparative studies [22] have indicated better antibacterial activity of Tetraclean. Although an antibiotic-containing RCI may have good short- and long-term effects, concerns have been expressed regarding the use of tetracycline (doxycycline) because of possible bacterial resistance to the antibiotic, allergy to doxycycline and tooth discoloration [23]. To overcome these concerns, Tetraclean NA was developed from the same manufacturer with antibiotic excluded from its formulation; which consists of citric acid, cetrimide and polypropylene glycol.

The purpose of this study was to determine the antimicrobial efficacy of different RCIs including NaOCl, Tetraclean and Hypoclean on root canals infected with *Enterococcus faecalis (E. faecalis)* before and after root canal preparation.

## Materials and Methods

*E. faecalis *(ATCC 29212) was used in this assay. The experimental suspensions were prepared by cultivating the biological indicator on the surface of brain-heart infusion (BHI) agar (Difco Laboratories, Detroit, MI, USA) and incubated at 37^°^C for 24 h. The bacterial cells were re-suspended in saline solution to reach a final concentration of about 3×10^8^ cells/mL adjusted to No.1 McFarland turbidity standard. The bacterial concentration was interpreted using an UV spectrophotometer (Model Nova 1600 UV, Piracicaba, SP, Brazil) regulated 600 nm wavelength.

Thirty-five extracted maxillary central incisors with intact cementum were removed from 0.2% thymol storage medium and were immersed in 5% NaOCl for 30 min to remove the organic tissues. The root canals were prepared using BioRace system (FKG Dentaire, La Chaux-de-Fonds, Switzerland) using BR0#25/0.08, BR1#15/0.05, BR2#25/0.04, BR3#25/0.06, BR4#35/0.04, BR5#40/0.04 and BR5C#40/0.02. The root canals were irrigated with 3 mL of 2.5% NaOCl between the instruments with an Ultradent syringe and 0.30-Navitip needle (Ultradent Products Inc., South Jordan, UT, USA). The crowns were removed and root lengths were standardized to 16 mm (from root apex to coronal border). Root canals were dried and filled with 17% EDTA for 3 min for smear layer removal. After root canal preparation, teeth were autoclaved for 30 min at 121^°^C temperature and 15 PSI pressure.

***Experimental strategy***

A split platform was used during inoculation with the bacteria [24]. Briefly, the coronal portion of the root canal was connected to the cut end of a 1.5 mL polypropylene Eppendorf tube using a cyanoacrylate adhesive. The entire surface of tooth-tube interface were coated with nail polish. The specimens were sterilized in 5% NaOCl for 30 min and then were placed in the BHI medium. To ensure disinfection, the test apparatus was incubated at 37^°^C for 24 h. Using sterilized syringes, the specimens were inoculated with *E. faecalis* every three days during a 60-day period (Figure 1). The teeth were kept in a humid environment at 37^°^C.

**Table 1:** Distribution of irrigants after root canal preparation

Groups	Methods	Culture
1	Hypoclean+Tetraclean NA	5
2	Hypoclean	5
3	Tetraclean NA	5
4	1% NaOCl+Tetraclean NA	5
5	1% NaOCl	5
6	Positive control	5

After contamination, the root canals were dried and refilled with sterile distilled water. Pre-instrumentation sample collection was conducted using three #40 paper points kept in each canal for 3 min. The points were then individually transported and immersed in 7 mL of BHI, with neutralizers (Lecithin, Tween 80 and sodium thiosulfate) at appropriate concentrations and incubated at 37^°^C for 48 h. 

After root canal preparation using the BioRace system (BR5C #40/0.02, BR6 #50/0.04 and BR7 #60/0.02), samples were randomly assigned to two positive and negative control groups and five experimental groups (*n*=5) according to the irrigation protocol: Hypoclean/Tetraclean NA; Hypoclean; Tetraclean NA; 1% NaOCl/Tetraclean NA and 1% NaOCl.

In the experimental groups, 20 min after irrigation with 3 mL of test RCIs, an additional irrigation with 5 mL of sterile distilled water was performed. The root canals were dried, filled with sterile distilled water, and then paper point sampling was done as described above. All samples were collected using three paper points. The points were individually transported, immersed in 7 mL of BHI with neutralizers (Lecithin, Tween 80 and sodium thiosulfate) at appropriate concentrations, and incubated at 37^°^C for 48 h. 

After 72 h, after the evaluation of changes in the culture medium, an inoculum of 0.1 mL from the medium was transferred to 7 mL of Letheen Broth (Difco Laboratories, Detroit, MI, USA) and incubated at 37^°^C for 48 h. The Gram staining of the BHI culture was used to confirm *E. faecalis* contamination. All the collections were carried out under aseptic conditions. Bacterial growth was analyzed by measuring the turbidity of the culture medium and then analyzed under UV spectrophotometry. The measurement of the optical density of culture medium was proportional to the number of present bacteria. For 60 days, five non-inoculated and five *E. faecalis *infected teeth were incubated at 37^°^C as negative and positive control samples, respectively.

***Statistical Analysis***

The mean and standard deviation of the optical density in the samples (pre-instrumentation, and 20 min and 72 h after irrigation) were obtained. The difference between groups was analyzed by ANOVA and post hoc Tukey’s tests using the SPSS software (SPSS version 20.0, SPSS, Chicago, IL, USA). The level of significance was set at 0.05.

## Results

The results showed presence of *E. faecalis* after the irrigation process in all of experimental groups, irrespective of the irrigating solution. The mean optical densities in both assessment periods showed significant reduction in bacterial count and significant differences between Hypoclean, Hypoclean/Tetraclean NA and 1% NaOCl (Tables 1 and 2). The mean values of optical density in positive and negative control samples were 0.601 and 0.000 nm, respectively.

## Discussion

This *in vitro* study compared the antimicrobial activity of different RCI with low surface tension including NaOCl, Tetraclean NA and Hypoclean on root canals infected with *E. faecalis *after variable time intervals.

*E. faecalis* was chosen for this study for some reasons. First, it is the most common bacterial species isolated from the failed endodontic treatments [25]. Second, it is a resistant microorganism that can grow in a monoculture [26]. Third, several studies have used it as the test organism [25, 26] and fourth, it is resistant to calcium hydroxide as the most common intracanal medicament [22, 25]. The reason for 60-day inoculation of *E. faecalis* suspension was to give time for complete penetration of the microorganisms into dentinal tubules [23].

According to previous classical studies, although mechanical instrumentation significantly reduces the microbial load of the root canal space, an appropriate root canal irrigant with antibacterial activity is required to enhance the disinfection process [3, 6]. 

One of the major drawbacks of NaOCl is its high surface tension [8] which limits the penetration of RCI into the irregularities of the root canal system. Adding surfactant to NaOCl enhances its penetration [9]. Hypoclean was constructed based on the very same concept. It has been revealed that Hypoclean was the most effective irrigant against *Candida albicans*, *Pseudomonas aeruginosa*, and *Lactobacillus casei*, using agar diffusion test [12]. Furthermore, using a human tooth model, Mohammadi *et al.* [9] demonstrated the substantivity of Hypoclean for up to 4 weeks. Considering the fact that NaOCl does not have substantivity, the substantivity of a NaOCl-based irrigants such as Hypoclean can be attributed to its deep penetration into irregularities of the root canal system, which makes chlorine ions available for a longer time. 

**Table 2:** Mean (SD) of optical densities (nm) in relation with the number of bacteria present in the experimental groups at three time intervals

**Groups **	**Before instrumentation**	**20 min after instrumentation**	**72 h after instrumentation**
**Hypoclean/Tetraclean NA**	0.605±0.033	0.017±0.014^a^*,*^b^	0.059±0.017^b^*, *c e
**Hypoclean**	0.539±0.040	0.014±0.023^a^*,*^b^	0.029±0.018^b^*, *d e
**Tetraclean NA**	0.659±0.168	0.305±0.109^b^	0.108±0.083^b^
**1% NaOCl/Tetraclean NA**	0.686±0.086	0.019± 0.020^a^*,*^b^	0.229±0.057^b^
**1% NaOCl**	0.569±0.065	0.025±0.032^a^*,*^b^	0.250±0.031^b^
**positive control**	0.634±0.149	0.634±0.149	0.652±0.160

a P<0.001 vs TetracleanNA;

b P<0.001 vs positive control;

c P<0.05 vs 1% NaOCl/Tetraclean NA;

d P<0.01 vs 1% NaOCl/Tetraclean NA;

e P<0.01 vs 1% NaOCl

Results of the present study showed that after Hypoclean, the best activity was attributed to Hypoclean/Tetraclean NA. Tetraclean NA has excluded antibiotic from its contents and is composed of citric acid and two detergents (propylene glycol and cetrimide). Presence of detergent can increases the penetration depth of Tetraclean. In a recent study, Poggio *et al.* [24] observed a significantly higher release of Ca^2+^ in samples submitted to citric acid based agents. The addition of cetrimide did not affect the chelating properties of EDTA and citric acid solutions because the concentration of Ca^2+^ released in the two solutions did not significantly differ [9, 24]. It indicates that, to obtain an efficient smear layer removal and to facilitate the biomechanical procedures, citric acid based agents can be applied and cetrimide can help in improving efficacy of the irrigating solutions. Moreover, cetrimide does not affect the demineralization ability of citric acid and EDTA solutions. 

Cationic surfactants are potent antimicrobial agents that have also been shown to act on biofilm components, but they have no decalcifying effects on root canal dentin [27]. EDTA and citric acid solutions are not effective against the biofilms at any tested concentration or time [28]; however, the antimicrobial activity of chelating agents in combination with cetrimide is greater than the use of chelating agents alone [29], and the combination of 0.2% cetrimide with either 15% EDTA or 15% citric acid gave 100% bacterial kill after 1 min of contact with the biofilms [30]. 

To improve their efficacy, root canal irrigants must be in contact with the dentin walls and debris [31]. The intimacy of this contact depends on the tendency of the irrigant to wet the solid dentin, and this property is strictly correlated to the surface tension of the irrigant [32]. Surface tension is defined as “the force between molecules that produces a tendency for the surface area of a liquid to decrease” [32]. RCI solutions should have very low surface tension. The wettability of the solution governs the capability of its penetration both into the main and lateral canals, and into the dentinal tubules [33]. By lowering the surface tension of RCI, their wettability improves and solutions modified with surfactants immediately spread on the dentin surface, yielding a zero-degree contact angle [34]. 

As Tetraclean is an antibiotic-based irrigation solution comprising of tetracycline isomer, there may be problems with tooth staining, bacterial resistance and sensitivity [35]. Using a solution without antibiotic as Tetraclean NA, could be a viable alternative. 

This *in vitro* study showed that Tetraclean NA keeps its antimicrobial activity on 60-day *E. faecalis *mature biofilms after 20 min and 72 h. Citric acid and cetrimide as two major components of Tetraclean NA, have antibacterial properties; however, the present study revealed that citric acid does demonstrate antimicrobial properties against anaerobic bacteria of the infected root canals, especially against *cocci *[36]. Further clinical and *in vitro* studies are needed to verify the antimicrobial effect of detergent-based irrigants with added surfactants in complex root canal system. 

## Conclusion

Under the experimental conditions, detergent-based irrigants can facilitate the biomechanical procedure of the irrigation solution. Moreover, the presence of cetrimide in the irrigating solutions may greatly assist in comprehensive antibacterial activity.
